# Supplementary material for: Do dogs eavesdrop on human interactions in a helping situation?
Source: PLoS One. 2020 Aug 26;15(8):e0237373. doi: 10.1371/journal.pone.0237373 (PMC7449479; doi:10.1371/journal.pone.0237373)
Supplement: S1 Table — (DOCX) [file pone.0237373.s001.docx]

**S1 Table. Individual characteristics of dogs.**

| **Name** | **Sex** | **Breed** | **Age (years)** | **Group** | **First test** | **Previous experience in other experiments** |
| --- | --- | --- | --- | --- | --- | --- |
| Albi | M | Czechoslovakian Wolfdog | 4 | Experimental | Impossible task | Yes |
| Arkani | M | Belgian Shepherd Dog (Tervueren) | 4 | Side control | Impossible task | No |
| Asha | F | Rhodesian Ridgeback | 1 | Experimental | Impossible task | No |
| Casey | F | Flat Coated Retriever | 4 | Side control | Impossible task | No |
| Cooper | M | Mixed breed | 3 | Experimental | Choice test | Yes |
| Crispy | F | Dalmatian | 10 | Side control | Choice test | Yes |
| Dagobert | M | Mixed breed | 3 | Side control | Impossible task | No |
| Denzel | M | American Staffordshire Terrier | 9 | Side control | Choice test | Yes |
| Eleanor | F | Mixed breed | 9 | Experimental | Impossible task | No |
| Emily | F | Border Collie | 9 | Experimental | Choice test | Yes |
| Flamme | M | Pyrenean Sheepdog | 10 | Side control | Choice test | Yes |
| Franzi | F | Mixed breed | 4 | Experimental | Impossible task | Yes |
| Gordon | M | Barzoi | 4 | Experimental | Choice test | No |
| Hailey | F | Golden Retriever | 5 | Experimental | Choice test | Yes |
| Holly | F | Mixed breed | 4 | Experimental | Impossible task | No |
| Hugo | M | Bernese Mountain Dog | 9 | Side control | Impossible task | Yes |
| Jace | M | Border Collie | 1 | Experimental | Impossible task | Yes |
| Jolie | F | Mixed breed | 8 | Side control | Choice test | Yes |
| Kimba | F | French Bulldog | 3 | Experimental | Choice test | No |
| Lenny | M | Canarian Warren Hound | 3 | Experimental | Choice test | Yes |
| Letti | F | Elo | 4 | Side control | Impossible task | Yes |
| Linus | M | Border Collie | 2 | Side control | Choice test | No |
| Lissy | F | White Swiss Shepherd Dog | 3 | Side control | Impossible task | Yes |
| Miley | F | Border Collie | 9 | Side control | Choice test | Yes |
| Milo | M | Labradoodle | 7 | Experimental | Choice test | No |
| Mira | F | Mixed breed | 4 | Experimental | Choice test | No |
| Molly | F | Mixed breed | 7 | Experimental | Choice test | No |
| Nina | F | Labrador Retriever | 9 | Side control | Choice test | No |
| Nuri | F | Mixed breed | 1 | Experimental | Impossible task | No |
| Orlando | M | Rough Collie | 7 | Side control | Choice test | Yes |
| Ruby | F | Mixed breed | 1 | Side control | Impossible task | Yes |
| Samson | M | American Staffordshire Terrier | 2 | Side control | Choice test | Yes |
| Santos | M | Boxer-Mix | 9 | Experimental | Impossible task | Yes |
| Sixtus | M | Brussels Griffon | 1 | Side control | Impossible task | Yes |
| Sokrates | M | Mixed breed | 12 | Experimental | Choice test | Yes |
| Tommy | M | Pembroke Welsh Corgi | 2 | Side control | Choice test | No |
| Ultimo | M | Border Collie | 8 | Experimental | Impossible task | Yes |
| Wasabi | F | Chihuahua-Mix | 5 | Experimental | Impossible task | Yes |
| Weichsel | F | Beagle | 12 | Experimental | Impossible task | No |
| Winston | M | Dachshund | 9 | Side control | Choice test | No |
| Yalla | F | Bergamasco Shepherd | 2 | Side control | Impossible task | Yes |
| Zuri | F | Rhodesian Ridgeback | 8 | Side control | Impossible task | Yes |
