# Supplementary material for: Do dogs eavesdrop on human interactions in a helping situation?
Source: PLoS One. 2020 Aug 26;15(8):e0237373. doi: 10.1371/journal.pone.0237373 (PMC7449479; doi:10.1371/journal.pone.0237373)
Supplement: S2 Table — Estimates, standard error, confidence intervals, results of significance tests and minimum and maximum of model estimates derived after excluding individuals one at a time. (DOCX) [file pone.0237373.s002.docx]

**S2 Table. Results of the full models in the impossible task.**

| **Response variable** | **Term** | **Estimate** | ***SE*** | **95% *CI*** | | ***z*** | ***p*** | **Min** | **Max** |
| --- | --- | --- | --- | --- | --- | --- | --- | --- | --- |
|  |  |  |  | **Lower** | **Upper** |  |  |  |  |
| Proportion of time dogs looked at the helpful experimenter | Intercept | -0.278 | 0.421 | -1.104 | 0.547 | -0.661 | .508 | -0.402 | -0.009 |
|  | Experimenter position | 0.423 | 0.566 | -0.685 | 1.532 | 0.749 | .454 | 0.157 | 0.711 |
|  | Test order | -0.189 | 0.576 | -1.318 | 0.941 | -0.327 | .743 | -0.520 | 0.016 |
|  | Experimenter position: Test order | 0.542 | 0.807 | -1.040 | 2.124 | 0.672 | .502 | 0.304 | 0.900 |
| Proportion of time dogs interacted with the helpful experimenter | Intercept | 0.237 | 0.400 | -0.547 | 1.021 | 0.593 | .553 | -0.080 | 0.479 |
|  | Experimenter position | 0.040 | 0.584 | -1.105 | 1.185 | 0.068 | .946 | -0.332 | 0.367 |
|  | Test order | -0.709 | 0.540 | -1.767 | 0.350 | -1.312 | .189 | -0.962 | -0.409 |
|  | Experimenter position: Test order | 0.283 | 0.782 | -1.249 | 1.815 | 0.362 | .718 | -0.033 | 0.666 |
| Proportion of time dogs spent close to the helpful experimenter | Intercept | 0.006 | 0.338 | -0.657 | 0.669 | 0.018 | .986 | -0.198 | 0.160 |
|  | Experimenter position | -0.505 | 0.468 | -1.423 | 0.413 | -1.079 | .281 | -0.701 | -0.193 |
|  | Test order | 0.120 | 0.445 | -0.752 | 0.991 | 0.269 | .788 | -0.190 | 0.326 |
|  | Experimenter position: Test order | 0.212 | 0.635 | -1.033 | 1.458 | 0.334 | .738 | -0.111 | 0.615 |

| **Response variable** | **Term** | **Estimate** | ***SE*** | **95% *CI*** | | **χ^2^** | **df** | ***p*** | **Min** | **Max** |
| --- | --- | --- | --- | --- | --- | --- | --- | --- | --- | --- |
|  |  |  |  | **Upper** | **Lower** |  |  |  |  |  |
| Proportion of time dogs gaze alternated to the helpful experimenter | Intercept | 0.109 | 0.270 | -0.482 | 0.639 |  |  |  | -0.147 | 0.241 |
|  | Experimenter position | -0.486 | 0.378 | -1.229 | 0.290 |  |  |  | -0.653 | -0.231 |
|  | Test order | 0.466 | 0.400 | -0.256 | 1.302 |  |  |  | 0.333 | 0.722 |
|  | Experimenter position: Test order | 0.006 | 0.570 | -1.142 | 1.064 | 0.000 | 1.000 | .991 | -0.249 | 0.173 |

Estimates, standard error, confidence intervals, results of significance tests and minimum and maximum of model estimates derived after excluding individuals one at a time.
